# Functional Status Enhances the FRAX^®^ Prediction of Fractures in Myasthenia Gravis: A 10-Year Cohort Study

**DOI:** 10.3390/jcm14093260

**Published:** 2025-05-07

**Authors:** Shingo Konno, Takafumi Uchi, Hideo Kihara, Hideki Sugimoto

**Affiliations:** Department of Neurology, Toho University Ohashi Medical Center, 2-22-36 Ohashi Meguro Ku, Tokyo 153-8515, Japan; takafumi.uchi@med.toho-u.ac.jp (T.U.); hideo.kihara@med.toho-u.ac.jp (H.K.); sugi-h@oha.toho-u.ac.jp (H.S.)

**Keywords:** myasthenia gravis, FRAX, fracture risk, functional status, activities of daily living, osteoporosis, bone mineral density, glucocorticoids, risk stratification, preventive care

## Abstract

**Background**: Patients with myasthenia gravis (MG) are susceptible to fractures due to glucocorticoid (GC) use and disease-related functional impairment affecting activities of daily living (ADL). The Fracture Risk Assessment Tool (FRAX^®^) estimates fracture probability but does not incorporate disease-specific functional status. We investigated whether combining FRAX^®^ with the Myasthenia Gravis Activities of Daily Living (MG-ADL) scale improves fracture risk stratification in MG patients. **Methods**: This single-center prospective cohort study followed 53 MG patients for 10 years (2012–2022) at Toho University Ohashi Medical Center, Japan. Patients were categorized into four groups based on baseline FRAX^®^ probability (calculated with bone mineral density [BMD]) and MG-ADL scores using median splits: high FRAX^®^/high MG-ADL (HH), high FRAX^®^/low MG-ADL (HL), low FRAX^®^/high MG-ADL (LH), and low FRAX^®^/low MG-ADL (LL). The primary outcome was incident major osteoporotic fracture (MOF). **Results**: Over 10 years, nine MOFs occurred: seven in the HH group (43.8%), two in the HL group (16.7%), and none in the LH or LL groups. Fracture-free survival differed significantly among the groups (log-rank *p* < 0.001), with the HH group exhibiting the lowest survival rate. Baseline characteristics, including age, disease duration, MG severity scores, BMD, and FRAX^®^ scores, differed significantly among groups. Specific MG-ADL items reflecting greater impairment (impairment of ability to arise from a chair, double vision, and ptosis) were significantly more pronounced in the HH group at baseline. **Conclusions**: Combining baseline FRAX^®^ scores with the MG-ADL assessment effectively stratifies long-term MOF risk in patients with MG. Individuals with both high FRAX^®^ and high MG-ADL represent a particularly high-risk subgroup. This dual-assessment approach may improve the identification of patients requiring targeted preventive interventions.

## 1. Introduction

Myasthenia gravis (MG) is an autoimmune disorder affecting neuromuscular junctions, leading to muscle weakness and fatigue [1]. Glucocorticoids (GCs) serve as a primary, often long-term, treatment for managing this condition [2]. However, the use of GCs is known to negatively impact bone health, as they can induce osteoporosis and consequently increase the risk of bone fractures [2]. Fractures represent a significant health complication that can impair function and quality of life (QOL), underscoring the importance of understanding and managing bone health specifically within the MG patient population, who face risks associated with both the disease and its treatment. Notably, previous research has indicated that osteoporotic fractures in MG patients are associated with the duration of glucocorticoid therapy, rather than the dose of prednisolone, and that glucocorticoid-induced osteoporosis (GCIO) aggravates the QOL in MG patients [3]. This further substantiates the importance of fracture risk management in the MG population. In addition to the effects of GC treatment, factors inherent to MG itself may influence fracture susceptibility. Reduced musculoskeletal fitness, stemming from the characteristic muscle weakness of MG, can lead to limitations in ADL, which is generally associated with adverse health outcomes, including falls and fractures [4]. Additionally, reduced physical activity leading to decreased mechanical loading [1,5,6], diminished sun exposure potentially lowering vitamin D levels [7], chronic inflammation inherent to MG’s autoimmune nature [8], and nutritional deficiencies or comorbid conditions [1,9] further complicate the skeletal risk profile in this population. Moreover, fractures themselves may impose a significant psychological burden, including fear of falling, anxiety, and depression, which can further erode overall well-being [10,11].

While FRAX^®^ is widely used to estimate fracture probability, its optimal application in patients with MG has been considered uncertain, possibly because standard risk factors do not fully encompass disease-specific aspects, like functional impairment [12]. Although the recent research indicates that FRAX^®^ can be a valuable tool for risk stratification within the MG population [12,13], it notably does not include direct measures of ADL. While both the MG-ADL and the Quantitative Myasthenia Gravis (QMG) score assess disease severity, the MG-ADL specifically focuses on the patient-reported impact of symptoms on daily activities, whereas the QMG provides a more objective, clinician-assessed measure of muscle strength deficits. Although correlated, they capture different facets of the disease burden. Therefore, understanding the combined influence of bone health status, as estimated by FRAX^®^, and MG-specific functional limitations is essential for potentially improving fracture risk prediction and guiding preventive strategies for these individuals. Given the potential influence of disease-specific functional limitations on fracture occurrence in MG, and the limitations of the existing risk assessment tools, like FRAX^®,^ in capturing this aspect, this study aimed to address this gap. Specifically, we investigated whether combining the FRAX^®^ score, representing general osteoporotic risk factors, with the MG-ADL scale, reflecting disease-specific functional status, could improve the prediction of major osteoporotic fractures in patients with MG. We conducted a prospective cohort study over a 10-year period, categorizing patients based on their baseline FRAX^®^ and MG-ADL scores [14], and compared subsequent fracture incidence among these groups.

## 2. Materials and Methods

### 2.1. Study Design and Setting

This investigation was conducted as a single-center, prospective cohort study. Participants were recruited from the Department of Neurology at Toho University Ohashi Medical Center, Tokyo, Japan. The observation period commenced with participant enrollment, which took place between April 2012 and July 2012, and continued until June 2022, spanning a total of 10 years.

### 2.2. Participants

Patients diagnosed with MG according to the established diagnostic criteria (e.g., clinical presentation, positive acetylcholine receptor antibody test, and/or characteristic electromyography findings) were eligible for inclusion. We excluded patients who were unable to provide informed consent, those with a history of major osteoporotic fracture prior to enrollment, or individuals with other known significant bone diseases (e.g., Paget’s disease and hyperparathyroidism) or conditions expected to severely impact bone metabolism or survival independently of MG or GC use. A total of 53 patients with MG fulfilled these criteria and were included in the final analysis.

### 2.3. Data Collection

Baseline data were collected at the time of enrollment (2012). This included demographic information (age, sex), clinical details related to MG (disease duration), MG subtype and severity assessed by the MG Foundation of America (MGFA) classification [15], MGFA post-intervention status [15], quantitative MG (QMG) score [15], MG composite (MGC) [16]), treatment history (especially GC use), bone mineral density (BMD) measurements, FRAX^®^ calculations, and MG-ADL scores. Participants were subsequently followed prospectively for 10 years (until June 2022). Information regarding the occurrence of new major osteoporotic fractures during the follow-up period was collected through patient self-reports during regular clinic visits and confirmed by a review of medical records and imaging reports, where available.

### 2.4. Measurements

*Fracture Risk Assessment Tool (FRAX^®^)*: The 10-year probability of major osteoporotic fracture (MOF; including clinical spine, hip, forearm, and humerus fractures) and hip fracture was calculated using the Japanese FRAX^®^ model. Calculations were performed both with and without baseline femoral neck BMD measurements. Adjustments for GC dose (prednisolone equivalent dose ≥5 mg/day for ≥3 months) were incorporated as per the standard FRAX^®^ guidelines.

*MG-ADL Scale*: Disease-specific functional status was assessed using the MG-ADL scale, an 8-item patient-reported outcome measure evaluating MG symptom severity related to daily activities. Scores ranged from 0 to 24, with higher scores indicating greater impairment [14].

*Fracture Outcome*: The primary outcome was the incidence of any new MOF occurring after baseline enrollment and during the 10-year follow-up period. Fractures were verified as described in the Data Collection Section.

*Bone Mineral Density (BMD)*: Areal BMD (g/cm^2^) of the lumbar spine (L1-L4) and femoral neck was measured at baseline using dual-energy X-ray absorptiometry (DEXA; GE Healthcare, Chicago, IL, USA).

*Other Variables*: Information on age, sex, body mass index (BMI), smoking status, alcohol intake, history of previous fractures (before MG diagnosis, if any), MG duration, cumulative GC dose (if available), and use of anti-osteoporosis medications was also collected at baseline.

### 2.5. Statistical Analysis

Participants were categorized into four groups based on baseline FRAX^®^ MOF probability (calculated with BMD) and MG-ADL scale scores, using the median values (9.0% and 2 points, respectively) as thresholds: high FRAX^®^/high MG-ADL (HH), high FRAX^®^/low MG-ADL (HL), low FRAX^®^/high MG-ADL (LH), and low FRAX^®^/low MG-ADL (LL). Baseline characteristics among the four groups were compared using Kruskal–Wallis tests for continuous variables and chi-squared tests or Fisher’s exact tests for categorical variables, as appropriate. Fracture-free survival curves were generated using the Kaplan–Meier method, and differences among the four groups were assessed using the log-rank test. Specific MG-ADL item scores potentially contributing to differences between groups were also explored descriptively or using appropriate non-parametric tests. All statistical analyses were performed with EZR (Saitama Medical Center, Jichi Medical University, Saitama, Japan), which is a graphical user interface for R (The R Foundation for Statistical Computing, Vienna, Austria). More precisely, it is a modified version of R commander designed to add statistical functions frequently used in biostatistics [17]. *p* < 0.05 was considered significant.

## 3. Results

### 3.1. Participant Characteristics

A total of 53 patients with MG were included in the analysis and categorized into four groups based on baseline FRAX^®^ MOF probability (with BMD) and MG-ADL scores: HH (*n* = 16), HL (*n* = 12), LH (*n* = 11), and LL (*n* = 14). Baseline characteristics of the participants stratified by these groups are presented in Table 1 and Table 2. Significant differences were observed among the groups at baseline for several characteristics. Notably, patients in the high FRAX^®^ groups (HH and HL) were significantly older than those in the low FRAX^®^ groups (LH and LL) (*p* < 0.001). Disease duration was significantly longer in the HH group compared to the LH and LL groups (*p* = 0.021). As per the grouping criteria, baseline MG-ADL total scores were significantly different across the four groups (*p* < 0.001), with HH and LH groups having higher scores than HL and LL groups. Similar significant differences were observed for the MGC score (*p* < 0.001), QMG score (*p* < 0.001), and the Japanese version of Myasthenia Gravis Quality of Life 15 (MG-QOL15-J) score [18] (*p* < 0.001), generally indicating greater disease severity and impact in the HH and LH groups compared to the HL and LL groups. Regarding bone health parameters (Table 2), baseline BMD and corresponding T-scores at both the lumbar spine and hip were significantly lower in the high FRAX^®^ groups (HH and HL) compared to the low FRAX^®^ groups (LH and LL) (all *p* ≤ 0.003). Consequently, calculated baseline FRAX^®^ probabilities for both MOF and hip fracture (with and without BMD) were significantly higher in the HH and HL groups compared to the LH and LL groups (all *p* < 0.001). The use of medications affecting bone turnover was significantly higher in the HH group compared to the LL group (*p* = 0.024). No significant differences were found among groups for baseline serum bone turnover markers.

### 3.2. Participant Flow/Follow-Up

**Figure 1:**
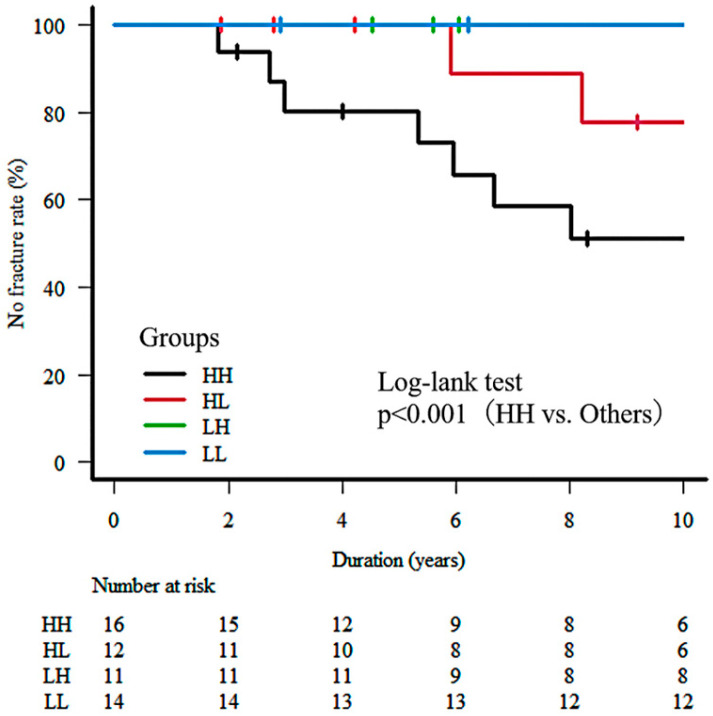
Kaplan–Meier analysis of fracture-free survival over 10 years stratified by baseline FRAX® MOF probability and MG-ADL score. Abbreviations: HH, high FRAX®/high MG-ADL group; HL, high FRAX®/low MG-ADL group; LH, low FRAX®/high MG-ADL group; LL, low FRAX®/low MG-ADL group.

All 53 participants were followed for up to 10 years. The number of patients remaining at risk at various time points throughout the follow-up period is shown in Figure 1.

The Kaplan–Meier curves show the cumulative incidence of remaining fracture-free (No fracture rate, %) over a 10-year follow-up period in four groups categorized according to baseline FRAX^®^ major osteoporotic fracture (MOF) probability (calculated with BMD) and MG-ADL scores. Participants were classified into four groups—HH, HL, LH, and LL—using the median values of FRAX^®^ MOF probability (≥9.0%) and MG-ADL score (≥2 points) as thresholds. The HH group (black line), representing participants with high FRAX^®^ MOF and high MG-ADL scores, exhibited significantly lower fracture-free survival compared to the other groups. Log-rank test revealed a significant difference (*p* < 0.001, HH vs. others). Tick marks indicate censored cases. The number at risk at each time point is shown below the *x*-axis.

### 3.3. Fracture Outcomes

During the 10-year follow-up period, a total of nine major osteoporotic fractures (MOFs) occurred. Seven fractures were observed in the HH (high FRAX^®^, high MG-ADL) group (7/16, 43.8%), and two fractures occurred in the HL (high FRAX^®^, low MG-ADL) group (2/12, 16.7%). No fractures were observed in the LH or LL groups (0/11 and 0/14, respectively) (Table 2, fracture-type breakdown and Figure 1). The types of fractures observed included lumbar vertebra (*n* = 4 in HH, *n* = 1 in HL), hip (*n* = 1 in HH, *n* = 1 in HL), thoracic vertebra (*n* = 1 in HH), and proximal humerus (*n* = 1 in HH) (Table 2). Kaplan–Meier analysis demonstrated distinct fracture-free survival curves among the four groups (Figure 1). The HH group exhibited the lowest fracture-free survival rate throughout the follow-up period. The HL group also showed a decline in fracture-free survival, although less pronounced than the HH group. The LH and LL groups remained fracture-free (100% survival). The log-rank test confirmed a statistically significant difference in fracture incidence among the four groups (*p* < 0.001).

### 3.4. MG-ADL Item Analysis

Analysis of individual baseline MG-ADL item scores revealed significant differences among the four groups for specific activities (Table 1). Notably, compared to the LL group (low FRAX^®^, low MG-ADL), the HH group reported significantly higher scores (indicating greater difficulty) for “Impairment of ability to arise from a chair” (*p* = 0.028), “Double vision” (*p* = 0.005), and “Eyelid droop” (ptosis) (*p* < 0.001). The LH group also showed significantly higher scores for “Double vision” and “Eyelid droop” compared to the LL group.

## 4. Discussion

This prospective cohort study demonstrated that combining baseline fracture risk assessment using FRAX^®^ with a measure of disease-specific functional status, the MG-ADL scale, effectively stratified the 10-year risk of major osteoporotic fractures in patients with MG. Our primary finding reveals a significantly elevated fracture incidence specifically concentrated within the subgroup of patients exhibiting both high FRAX^®^ scores and high MG-ADL scores at baseline. Furthermore, specific functional limitations captured by the MG-ADL scale, such as an impairment of the ability to arise from a chair and prominent ocular symptoms, were identified as characteristic baseline features of this highest-risk group. These findings directly address the research objective of exploring whether incorporating ADL measures can enhance fracture risk prediction beyond traditional risk factors alone in the MG population.

Our demonstration that combining baseline FRAX^®^ probability with the MG-ADL score effectively stratifies 10-year fracture risk significantly advances risk assessment in patients with MG. While the FRAX^®^ tool is established for integrating multiple clinical risk factors, with or without BMD, to estimate fracture probability in general populations [19], it does not account for disease-specific factors, such as muscle weakness or functional impairments. In our study, although groups with high FRAX^®^ scores (HH and HL) included all observed fractures, the marked divergence between the HH group (43.8% fracture rate) and the HL group (16.7% fracture rate), underscores the substantial additional risk associated with poor baseline functional status. This emphasizes the limitations of FRAX^®^ when used alone in chronic neurological diseases, like MG-ADL. This occurs where specific impairments substantially influence fall and fracture risks.

The necessity of considering factors beyond standard FRAX^®^ inputs resonates with the findings in other chronic conditions, for instance, in chronic obstructive pulmonary disease patients, FRAX^®^ alone may underestimate risk by neglecting disease-related factors, such as an increased propensity for falls [20]. Similarly, in MG, higher MG-ADL scores likely reflect greater general frailty and vulnerability to falls, elevating fracture risk independently of FRAX^®^. Our findings strongly suggest that assessing MG-ADL alongside FRAX^®^ is crucial for accurately identifying the subset of MG patients at the highest fracture risk, allowing for more targeted and effective preventative interventions. This conclusion aligns with the prior research showing that osteoporotic fractures in MG patients were associated with the total quantitative MG score [3], suggesting that functional impairment due to the disease contributes to fracture vulnerability. To further support the construct validity of the MG-ADL scale as a marker of functional impairment, we confirmed a significant positive correlation between MG-ADL and QMG scores at baseline (Figure S1). Furthermore, the same study reported that GCIO worsens QOL as measured by the MG-QOL15 score, which aligns with our study’s finding that the high-risk group (HH) had poorer baseline QOL scores.

Beyond the overall MG-ADL score, our analysis highlights that specific item—particularly impairment of the ability to arise from a chair, double vision, and eyelid droop—were more pronounced at baseline in the high-risk HH group. Among these, impairment of the ability to arise from a chair directly reflects proximal limb muscle weakness, a well-established risk factor for falls and subsequent fractures across various populations, and likely contributed substantially to the vulnerability of the HH group. The association with ocular symptoms (double vision and ptosis) may also indirectly increase fall risk by impairing visual cues critical for maintaining balance and navigating environments safely. Thus, these functional impairments align not only signal more severe MG pathology but also with broader concepts of frailty, where muscle weakness and reduced physical activity predict adverse outcomes, including falls, fractures, and hospitalization in older adults [21].

Furthermore, the MG-ADL scale is crucial in defining treatment success. Achieving “minimal symptom expression” (MSE) [22]—typically defined by an MG-ADL total score of 0 or 1—represents the near-complete resolution of symptoms affecting daily life and has emerged as a key therapeutic target. Reaching this goal, alongside the related target of treatment goal, like achieving a QOL-optimized minimal manifestation status with low-dose prednisolone [23], underscores the clinical importance of assessing and improving functional status. Achieving better symptom control and functional improvement not only enhances QOL but, as suggested by our study, possibly through various mechanisms may also reduce fracture risk by lowering fall risk susceptibility. Therefore, the elevated scores on these specific MG-ADL items in the HH group likely signal not only substantial underlying bone fragility (indicated by high FRAX^®^) but also heightened physical vulnerability due to MG-related impairment, explaining the markedly increased fracture incidence observed.

The findings of this study carry significant implications for the clinical management of patients with MG and future research directions. Routine incorporation of disease-specific functional status assessment, such as the MG-ADL scale, alongside standard fracture risk evaluations, like FRAX^®^, would enable clinicians to move beyond generic risk prediction and identify a particularly vulnerable subgroup (high FRAX^®^ and high MG-ADL) warranting prioritized bone-protective therapies and targeted fall-prevention strategies, consistent with the recommendations for managing GCIO [24]. Recognizing bone health as a critical, yet often overlooked, comorbidity in MG is essential. Furthermore, interventions aimed at improving functional status, such as tailored physical therapy programs, which have shown promise in improving ADL scale in MG patients, could play a pivotal role in mitigating fracture risk, representing an important avenue for future investigation. Consequently, in MG management, maintaining bone health and improving functional status should be prioritized alongside symptom control to achieve long-term QOL maintenance and fracture prevention. Future research should focus on validating this combined risk stratification approach in larger and more diverse MG cohorts, establishing specific intervention thresholds, and exploring cost-effectiveness and investigating the mechanisms by which specific MG-related impairments, such as ocular symptoms, influence fall, and fracture risk, occur. Ultimately, this study underscores the multifactorial nature of fracture risk in chronic neurological diseases, arising both from general factors, such as age and bone mineral density, and from disease-specific factors, such as functional impairments. While FRAX^®^ incorporates key risk factors, including age, chronic glucocorticoid use, and optionally BMD, it does not capture the disease-specific functional impairment inherent to MG. Our findings demonstrate that the MG-ADL score provides this crucial complementary information, likely reflecting increased fall risk and frailty not accounted for by FRAX^®^ alone, thereby significantly enhancing risk stratification. It highlights the critical need for personalized risk assessments that explicitly integrate both dimensions to optimize patient care.

## 5. Conclusions

This study provides compelling evidence that integrating a disease-specific functional measure, the MG-ADL scale, with the established FRAX^®^ tool significantly enhances the stratification of long-term fracture risk in patients with MG. We identified a distinct subgroup characterized by both high baseline FRAX^®^ scores and high MG-ADL scores who face a markedly elevated risk of MOF, highlighting the synergistic impact of underlying bone fragility and functional impairment.

While these findings underscore the potential clinical utility of this combined assessment approach for personalizing preventive strategies, several important limitations should be acknowledged. Most notably, our study’s small sample size (*n* = 53) divided into four groups results in limited statistical power, which affects the robustness of our findings. The single-center design with exclusively Japanese participants significantly restricts the generalizability of our results to different ethnic populations, healthcare systems, and clinical settings, where MG management practices may differ.

Future research should focus on validating these findings in larger, diverse, multi-center prospective cohorts with adequate statistical power to confirm the predictive value of combined FRAX^®^ and MG-ADL assessment. Studies should comprehensively address the mechanisms by which specific functional impairments, including ocular symptoms and proximal limb weakness, contribute to fall and fracture risk in MG. Investigations should also explore targeted intervention strategies to mitigate these risks, validate the combined FRAX^®^ and MG-ADL assessment approach across diverse cohorts, and ultimately establish optimized preventive frameworks tailored to MG-specific vulnerabilities. Furthermore, future studies should evaluate potential ethnic and regional variations in these relationships and assess the efficacy and cost-effectiveness of targeted interventions in the identified high-risk population. Despite these limitations, this study offers a valuable, practical step toward improving the identification and management of fracture risk in patients living with MG.

## Figures and Tables

**Table 1:** Patient characteristics and treatments for myasthenia gravis.

	HH	HL	LH	LL	*p* Value
	*n* = 16	*n* = 12	*n* = 11	*n* = 14	
Onset age of myasthenia gravis (MG), (years)	47.0 [36.5, 62.5]	49.5 [45.5, 63.0]	36.0 [23.50, 44.5]	43.5 [34.5, 49.7]	0.106
Disease duration, years	24.0 [8.0, 31.2] ***,†††	12.0 [5.5, 26.0] **,††	7.00 [4.0, 14.5]	4.5 [2.6, 10.7]	0.021
Subtype (*n*, %)					
Ocular MG	1 (6.2)	5 (41.7)	3 (27.3)	6 (42.9)	0.484
Generalized early-onset MG	7 (43.8)	5 (41.7)	6 (54.5)	4 28.6)	
Generalized late-onset MG	4 (25.0)	1 (8.3)	0 (0.0)	1 (7.1)	
Generalized thymoma-associated MG	2 (12.5)	1 (8.3)	1 (9.1)	2 (14.3)	
Generalized muscle-specific kinase antibody positive MG	0 (0.0)	0 (0.0)	0 (0.0)	0 (0.0)	
Generalized antibody-negative MG	2 (12.5)	0 (0.0)	1 (9.1)	1 (7.1)	
Myasthenia Gravis Foundation of America classification at worst (*n*, %)					
Class I	2 (12.5)	5 (41.7)	2 (18.2)	7 (50.0)	0.186
Class II	4 (25.0)	4 (33.3)	5 (45.5)	6 (42.9)	
Class III	5 (31.2)	0 (0.0)	2 (18.2)	1 (7.1)	
Class IV	3 (18.8)	0 (0.0)	2 (18.2)	0 (0.0)	
Class V	2 (12.5)	3 (25.0)	0 (0.0)	0 (0.0)	
Myasthenia Gravis Foundation of America post-intervention status (*n*, %)					
Complete stable remission	0 (0.0) ‡	1 (8.3) †	0 (0.0)	3 (21.4)	0.024
Pharmacological remission	0 (0.0)	1 (8.3)	0 (0.0)	2 (14.3)	
Improved	12 (75.0)	5 (41.7)	6 (54.5)	4 (28.6)	
Minimal Manifestations	3 (18.8)	5 (41.7)	1 (9.1)	4 (28.6)	
Unchanged	1 (6.2)	0 (0.0)	4 (36.4)	1 (7.1)	
Myasthenia gravis activities of daily living scale (point)					
Total	2.5 [2.0, 3.2] ***,‡‡‡	1.0 [0.0, 1.0]	3.0 [3.0, 4.0] †††,***	1.0 [0.0, 1.0]	<0.001
Talking	0.0 [0.0, 0.0]	0.0 [0.0, 0.0]	0.0 [0.0, 0.0]	0.0 [0.0, 0.0]	0.545
Chewing	0.0 [0.0, 0.0]	0.0 [0.0, 0.0]	0.0 [0.0, 0.0]	0.0 [0.0, 0.0]	0.551
Swallowing	0.0 [0.0, 0.0]	0.0 [0.0, 0.0]	0.0 [0.0, 0.0]	0.0 [0.0, 0.0]	0.157
Breathing	0.0 [0.0, 0.0]	0.0 [0.0, 0.0]	0.0 [0.0, 0.0]	0.0 [0.0, 0.0]	0.223
Brushing teeth or hair	0.5 [0.0, 1.0]	0.0 [0.0, 0.0]	0.0 [0.0, 1.5]	0.0 [0.0, 0.0]	0.052
Arising from chair	0.5 [0.0, 1.0] *,‡‡	0.0 [0.0, 0.0]	0.0 [0.0, 1.5]	0.0 [0.0, 0.0]	0.028
Double vision	0.5 [0.0, 1.0] †	0.0 [0.0, 0.0]	1.0 [0.0, 1.5] ‡‡	0.0 [0.0, 0.0]	0.005
Eyelid droop	1.0 [1.0, 2.0] ‡	0.0 [0.0, 1.0]	1.0 [1.0, 2.5] **	0.0 [0.0, 1.0]	<0.001
Myasthenia gravis composite (point)	5.0 [3.5, 7.2] *,‡‡	0.5 [0.0, 1.2]	4.0 [2.5, 5.5] ‡‡	0.0 [0.0, 2.5]	<0.001
Quantitative myasthenia gravis score (point)	6.0 [4.7, 8.5] *,‡‡	2.5 [1.7, 3.2]	6.0 [4.5, 8.0] *, ‡‡	2.0 [2.0, 3.0]	<0.001
Myasthenia gravis quality of life 15 (point)	24.0 [14.0, 30.7] *,‡‡	5.5 [1.7, 14.2]	22.0 [15.0, 39.0] *,‡‡	3.5 [1.2, 7.7]	<0.001
Treatment for myasthenia gravis					
Prednisolone use (*n*, %)	15 (93.8)	9 (75.0)	9 (81.8)	8 (57.1)	0.117
Current dose of prednisolone (mg/day)	2.0 [0.0, 5.5]	1.5 [0.0, 3.5]	2.0 [1.5, 7.5]	5.5 [4.0, 13.6]	0.182
Max dose of prednisolone (mg/day)	45.0 [37.5, 50.0]	35.0 [20.0, 50.0]	50.0 [30.0, 50.0]	40.0 [33.7, 46.2]	0.882
Duration of prednisolone treatment (years)	9.0 [3.4, 14.5]	4.0 [2.5, 13.0]	6.0 [2.7, 7.0]	3.7 [2.6, 11.0]	0.833
Total prednisolone dose within 1-year period (g/year)	0.9 [0.04, 1.3]	0.4 [0.01, 1.1]	0.7 [0.5, 1.7]	2.2 [0.2, 4.6]	0.498
Calcineurin inhibitor use (*n*, %)	10 (62.5)	6 (50.0)	5 (45.5)	3 (21.4)	0.155
Current dose of tacrolimus (mg/day)	3.0 [3.0, 3.0]	2.0 [1.5, 2.2]	1.5 [0.0, 3.2]	3.0 [3.0, 3.0]	0.477
Current dose of cyclosporine (mg/kg/day)	2.2 [1.5, 3.0]	2.4 [2.4, 2.4]	4.5 [4.1, 4.7]	1.0 [0.5, 1.5]	0.117

NOTE: Comparisons among the four groups (HH, HL, LH, LL) were performed using the Kruskal–Wallis test for continuous variables and the chi-squared test or Fisher’s exact test for categorical variables, as appropriate (*p*-values shown in the last column). Symbols indicate statistically significant differences in post hoc pairwise comparisons (Mann–Whitney U test with Bonferroni correction): *** *p* < 0.001 vs. HL; ** *p* < 0.01 vs. HL; * *p* < 0.05 vs. HL. ††† *p* < 0.001 vs. LH; †† *p* < 0.01 vs. LH; † *p* < 0.05 vs. LH. ‡‡‡ *p* < 0.001 vs. LL; ‡‡ *p* < 0.01 vs. LL; ‡ *p* < 0.05 vs. LL. Abbreviations: HH, high FRAX^®^/high MG-ADL group; HL, high FRAX^®^/low MG-ADL group; LH, low FRAX^®^/high MG-ADL group; LL, low FRAX^®^/low MG-ADL group.

**Table 2:** Parameters related to bone metabolism and FRAX^®^ score.

	HH	HL	LH	LL	*p* Value
	*n* = 16	*n* = 12	*n* = 11	*n* = 14	
Medication of bone turnover (*n*, %)	14 (87.5) ‡‡	9 (75.0)	7 (63.6)	5 (35.7)	0.024
Bisphosphates	13 (92.8)	9 (100.0)	6 (66.7)	4 (80.0)	0.165
Vitamin D	0 (0.0)	0 (0.0)	1 (11.1)	1 (20.0)	0.265
others (%)	1 (6.2)	0 (0.0)	0 (0.0)	0 (0.0)	0.324
Serum bone isoform of alkaline phosphatase (μg/L)	13.0 [9.7, 15.5]	12.0 [10.5, 15.0]	10.0 [9.0, 15.0]	10.0 [7.2, 13.0]	0.436
Serum pyridinoline cross-linked aminoterminal telopeptide of type I collagen (nmol BEC/L)	13.5 [11.7, 17.8]	12.9 [9.4, 14.1]	12.3 [8.2, 16.4]	11.8 [10.4, 12.7]	0.412
Bone mineral density (BMD) of lumbar (g/cm^2^)	0.72 [0.62, 0.79] †,‡	0.74 [0.62, 0.82] ‡	0.87 [0.82, 1.03]	0.88 [0.82, 0.95]	0.001
Bone mineral density of hip (g/cm^2^)	0.56 [0.49, 0.69] †,‡‡	0.54 [0.51, 0.63] ††,‡‡	0.77 [0.67, 0.82]	0.73 [0.67, 0.76]	<0.001
T-score of bone mineral density of lumbar	−2.4 [−3.0, −1.7] †, ‡	−2.3 [−3.0, −1.3]	−1.3 [−1.4, −0.6]	−0.8 [−1.5, 0.3]	0.003
T-score of bone mineral density of hip	−2.0 [−2.5, −1.2] ††,‡‡	−2.3 [−2.5, −1.6] †,‡	−0.6 [−1.0, −0.2]	−0.9 [−1.0, −0.2]	<0.001
Factors of FRAX calculation					
Age (years)	69.5 [66.0, 76.0] †††,‡‡‡	64.0 [63.5, 74.2] ††,‡‡	47.0 [42.0, 57.5]	48.5 [45.2, 56.5]	<0.001
Sex, Female (%)	15 (93.8)	10 (83.3)	7 (63.6)	10 (71.4)	0.229
Body mass index (kg/m^2^)	21.5 [20.2, 23.5]	21.7 [20.3, 24.7]	20.9 [19.5, 22.9]	21.5 [20.7, 24.0]	0.791
Previous fracture (*n*, %)	1 (6.2)	0 (0.0)	0 (0.0)	0 (0.0)	0.502
Parent’s hip fracture (*n*, %)	2 (12.5)	1 (8.3)	0 (0.0)	0 (0.0)	0.382
Current smoking (*n*, %)	0 (0.0)	1 (8.3)	2 (18.1)	0 (0.0)	0.055
Alcohol intake (>3 units/day) (*n*, %)	0 (0.0)	0 (0.0)	0 (0.0)	0 (0.0)	NA
Glucocorticoid use (>5 mg/day of PSL or equivalent for >3 months) (*n*, %)	12 (75.0)	9 (75.0)	9 (81.8)	8 (57.1)	0.538
Rheumatoid arthritis (*n*, %)	2 (12.5)	0 (0.0)	0 (0.0)	0 (0.0)	0.187
Secondary osteoporosis (*n*, %)	0 (0.0)	0 (0.0)	0 (0.0)	0 (0.0)	NA
Hip fracture risk with BMD (%)	5.3 [1.9, 11.5] †††,‡‡‡	3.1 [2.0, 6.9] †††,‡‡	0.2 [0.1, 0.6]	0.3 [0.1, 0.5]	<0.001
Hip fracture risk without BMD (%)	6.1 [4.2, 13.7] †††,‡‡‡	2.0 [1.3, 6.7]	0.3 [0.1, 0.7]	0.2 [0.1, 0.5]	<0.001
Major osteoporotic fracture risk with BMD (%)	17.0 [12.0, 32.2] †††,‡‡‡	12.0 [10.0, 19.5] †††,‡‡‡	3.2 [1.8, 4.2]	3.5 [2.1, 5.4]	<0.001
Major osteoporotic fracture risk without BMD (%)	18.0 [14.0, 27.2] †††,‡‡‡	11.0 [8.7, 18.2] †††,‡‡‡	2.4 [1.5, 4.8]	3.0 [2.0, 6.1]	<0.001
Major osteoporotic fracture within 10 years (*n*, %)	8 (50.0)	2 (16.6)	0 (0.0)	0 (0.0)	
Hip fracture (*n*, %)	1 (6.2)	1 (8.3)			
Lumbar vertebra fracture (*n*, %)	5 (31.2)	1 (8.3)			
Thoracic vertebra fracture (*n*, %)	1 (6.2)	0 (0.0)			
Proximal humerus fracture (*n*, %)	1 (6.2)	0 (0.0)			

NOTE: Comparisons among the four groups (HH, HL, LH, LL) were performed using the Kruskal–Wallis test for continuous variables and the chi-squared test or Fisher’s exact test for categorical variables, as appropriate (*p*-values shown in the last column). Symbols indicate statistically significant differences in post hoc pairwise comparisons (Mann–Whitney U test with Bonferroni correction): ††† *p* < 0.001 vs. LH; †† *p* < 0.01 vs. LH; † *p* < 0.05 vs. LH. ‡‡‡ *p* < 0.001 vs. LL; ‡‡ *p* < 0.01 vs. LL; ‡ *p* < 0.05 vs. LL. Abbreviations: HH, high FRAX^®^/high MG-ADL group; HL, high FRAX^®^/low MG-ADL group; LH, low FRAX^®^/high MG-ADL group; LL, low FRAX^®^/low MG-ADL group; NA, not avirable.

## Data Availability

The data are not publicly available because they contain information that can compromise the privacy of the research patients. The data supporting the findings of this study are available, except for personal patient information, upon request from the corresponding authors.

## Supplementary Materials

The following supporting information can be downloaded at: https://www.mdpi.com/article/10.3390/jcm14093260/s1, Figure S1: Correlation between MG-ADL and QMG scores at baseline (*n* = 53).
