# Supplementary material for: Functional Status Enhances the FRAX® Prediction of Fractures in Myasthenia Gravis: A 10-Year Cohort Study
Source: J Clin Med. 2025 May 7;14(9):3260. doi: 10.3390/jcm14093260 (PMC12072394; doi:10.3390/jcm14093260)
Supplement: Supplementary file 1 [file jcm-14-03260-s001.zip › jcm-3610472-supplementary.pdf]

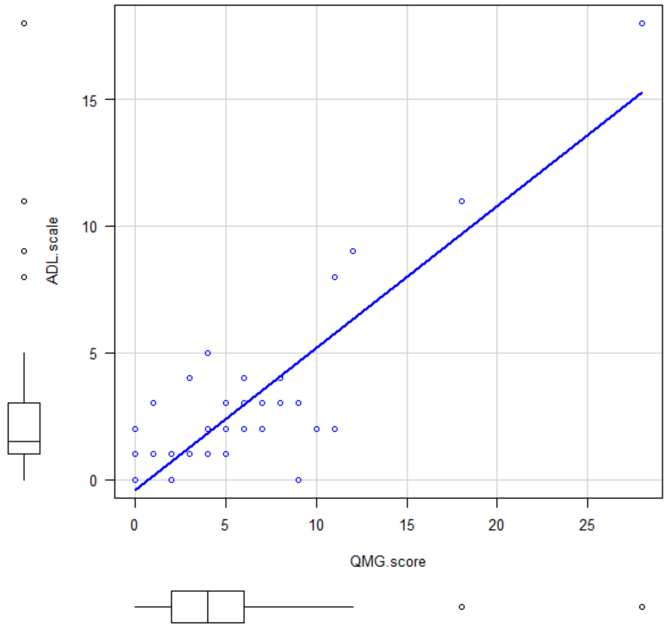

Figure S1. Correlation between MG-ADL and QMG scores at baseline (n = 53). A significant positive association was observed (Spearman's $\rho = 0.865$, $p < 0.001$), supporting the construct validity of the MG-ADL scale as a measure of functional impairment in MG.
